# Biventricular Cardiac Resynchronization Therapy with Atrial Sensing but No Atrial Lead: A Prospective Registry of Patients, Complications, and Therapy Responses

**DOI:** 10.3390/jcm14145009

**Published:** 2025-07-15

**Authors:** Christof Kolb, Endre Zima, Martin Arnold, Marián Fedorco, Hendrik Bonnemeier, Thomas Deneke, Burghard Schumacher, Peter Nordbeck, Clemens Steinwender, Theresa Storz, Béla Merkely, Lars Anneken, Angelika Felk, Carsten Lennerz

**Affiliations:** 1 Department of Cardiology, German Heart Centre, TUM University Hospital, Technical University of Munich, 80636 Munich, Germanylennerz@dhm.mhn.de (C.L.); 2 Department of Cardiology, Rhythmology, Angiology and Intensive Care Medicine, Heart Centre Osnabrück, Hospital Osnabrück, Westphalian Wilhelms University of Münster, 49076 Osnabrück, Germany; 3 Heart and Vascular Center, Semmelweis Medical University, 1122 Budapest, Hungary; 4 Department of Cardiology, Friedrich-Alexander-Universität Erlangen-Nuremberg, 91054 Erlangen, Germany; 5 Department of Cardiology, University Hospital Olomouc, 77900 Olomouc, Czech Republic; 6 Medical Faculty, Christian-Albrechts-University, 24105 Kiel, Germany; 7 Heart Center Bad Neustadt, 97616 Bad Neustadt, Germany; 8 2nd Department of Medicine, Westpfalz-Klinikum, 67655 Kaiserslautern, Germany; 9 Internal Medicine I, University Hospital Würzburg, 97080 Würzburg, Germany; 10 Department of Cardiology, University Hospital, Medical Faculty, Johannes Kepler University, 4020 Linz, Austria; clemens.steinwender@kepleruniklinikum.at; 11 Biotronik, 12359 Berlin, Germany; angelika.felk@biotronik.com; 12 Partner Site Munich Heart Alliance, German Centre for Cardiovascular Research (DZHK), 80636 Munich, Germany

**Keywords:** cardiac resynchronization therapy, two-lead CRT-D, CRT-DX, safety, patient selection, atrial floating sensing dipole

## Abstract

**Background/Objectives:** Patients with normal sinus rhythms undergoing cardiac resynchronization therapy defibrillator (CRT-D) implantation may benefit from a novel two-lead CRT-D system (CRT-DX), which features an atrial sensing dipole integrated into the right ventricular lead. This single-arm, international, non-controlled investigation focused on the safety and clinical efficacy of CRT-DX devices in CRT-D candidates who do not require atrial pacing. **Methods:** Patients indicated for CRT-D implantation (resting heart rates > 40 bpm and ≥100 bpm during exercise, no second or higher-degree AV block, and no history of persistent or permanent atrial fibrillation) were enrolled across 21 sites in four European countries. The primary endpoint was the need for an additional RA lead implantation within 12 months. Secondary endpoints comprised any invasive re-intervention to the CRT-DX system or infection. **Results:** Among the 110 patients (mean age 62 years, 70% male), 60% had an underlying non-ischemic cardiac disease. During 12 months of follow-up, RA lead implantation was required in two patients for atrial undersensing or chronotropic incompetence (RA lead implantation-free rate: 98.2% (95% CI: 92.7–99.5%)). Atrial sensing amplitudes were stable (mean: 4.7 ± 1.7 mV), AV-synchrony was maintained at >99%, and the median percentage of biventricular pacing exceeded 98%. The left ventricular ejection fraction improved by an absolute 14.7%. **Conclusions:** Using simple, clinically applicable inclusion criteria, the two-lead CRT-DX system demonstrated a low rate of subsequent RA lead implantations (1.8%) and maintained adequate RA sensing amplitudes throughout the observation period. The two-lead CRT-DX concept appears to be a feasible alternative for patients with preserved chronotropic competence.

## 1. Introduction

Cardiac resynchronization therapy (CRT) with biventricular pacing is an established treatment for patients with a wide QRS complex and severe, symptomatic heart failure despite optimal pharmacological therapy. CRT benefits are strongly supported by current clinical guidelines [1,2] and robust evidence from randomized trials, which consistently demonstrate reductions in morbidity and mortality compared to drug therapy alone [3,4,5]. Conventional CRT or CRT defibrillator (CRT-D) treatment requires a complex implanted system with three leads and is thus associated with a relevant rate of lead-related complications. Comparisons between single- and dual-chamber implantable cardioverter-defibrillators suggest that complication rates increase with the number of leads [6,7]. A simplification of the CRT-D system may therefore represent a strategy for reducing the complication rates in CRT-D recipients.

A CRT-D system with two leads (the CRT-DX concept) has recently been introduced, in which atrial sensing is provided by two rings placed on the right ventricular (RV) lead at 15 or 17 cm (center of the dipole) from the lead tip. Advanced signal processing by the CRT-DX electronic circuitry, including up to four-fold amplification of the atrial signal, enables adequate atrial sensing even in patients with long-lasting or permanent atrial fibrillation, but the system is not designed to deliver atrial pacing [8,9]. RV and left ventricular (LV) sensing and pacing in CRT-DX devices are performed using the same method as conventional three-lead CRT-D systems.

A potential disadvantage of the CRT-DX concept is the need for late atrial lead implantation in cases of new-onset bradycardia or loss of atrial sensing due to a less stable position of the free-floating atrial dipole. Moreover, there is a lack of standardized criteria for identifying and excluding CRT-DX candidates who might later develop the need for bradycardia pacing.

Clinical data regarding the use of CRT-DX in patients with CRT-D indication and sinus rhythms is still limited [10,11]. The prospective BIO|REDUCE study was designed to evaluate the performance of the CRT-DX system in another population of CRT-D candidates selected using clinically straightforward criteria and treated according to the standard care. The study aimed to determine whether CRT-DX implantation results in a re-intervention rate for atrial leads comparable to that of conventional CRT-D systems and whether the CRT response, facilitated by preserved atrioventricular (AV) synchrony and a high proportion of biventricular pacing, is similar to that of standard CRT-D systems.

## 2. Methods

### 2.1. Study Design and Conduction

BIO|REDUCE was a prospective, single arm, non-controlled, post-market observational study (ClinicalTrials.gov Identifier: NCT03839121) conducted at 21 sites in four European countries and in accordance with the Declaration of Helsinki. The study protocol was approved by the ethics committees and authorities for all participating sites according to the country-specific requirements. All patients provided written informed consent before enrollment.

### 2.2. Patient Enrollment and Follow-Up

Patients were recruited between July 2019 and June 2022. After CRT-DX implantation, they were followed for 12 months through on-site visits at 3, 6, and 12 months. Continuous remote monitoring (Home Monitoring®, Biotronik SE & Co. KG, Berlin, Germany) was applied in all patients.

Inclusion criteria were age ≥ 18 years and an indication for CRT-D implantation according to the ESC Guidelines [12,13]. In addition, the patients had to be in New York Heart Association (NYHA) class II or class III despite optimized pharmacological heart failure therapy and to be in sinus rhythm without a history of persistent or permanent atrial fibrillation. The required sinus rates were >40 bpm at rest and ≥100 bpm during exercise (tested within 3 months before enrollment). Exclusion criteria were (i) sinus node dysfunction or other anticipated circumstances requiring atrial pacing, (ii) the second- or third-degree AV block, or (iii) a premature ventricular contraction rate above 5%.

### 2.3. CRT-DX System

All patients were implanted with a commercially available CRT-D device (Ilivia, Inlexa, Intica, Rivacor, or Acticor, manufactured by Biotronik SE & Co. KG, Berlin, Germany), a compatible RV defibrillation lead with atrial dipole (RV-DX lead, models Plexa ProMRI S DX 65/15 or 65/17; Biotronik SE & Co. KG, Berlin, Germany), and any LV lead (type and manufacturer freely selected by the implanter). According to the study protocol, implantation of an RV-DX lead was not recommended if the atrial sensing amplitude measured with an external pacing system analyzer during implantation was below 1.0 mV. This cutoff value was based on recommendations derived from an expert survey.

### 2.4. Rationale and Study Endpoints

The implantation of a CRT-DX system may be beneficial if the rate of right atrial (RA) lead implantations in patients with initial CRT-DX implantation is lower than the re-intervention rate on the atrial lead in a three-lead CRT-D system. Therefore, the implantation of an RA lead for any clinical reason after a successfully concluded initial CRT-DX implantation was defined as the primary endpoint. The three secondary endpoints were any post-implant surgical re-intervention involving (i) any component of the CRT-DX system, (ii) any lead complication, and (iii) device or pocket infection. Additionally, atrial sensing performance and clinical outcome, measured by left ventricular ejection fraction (LVEF) and NYHA class, were assessed at baseline and at 12 months.

### 2.5. Statistical Methods

For sample size planning, a dislocation rate of RA leads in CRT-D systems of 3% was assumed [14,15,16,17,18]. Assuming a similar implantation rate of RA leads in the CRT-DX system, a sample size of at least 100 patients was calculated to achieve a 95%, 80%, or 58% probability of observing at least 1, 2, or 3 primary endpoints, respectively [19].

Patients in whom implantation of any component of the CRT-DX system failed or in whom the implanter chose to switch to the conventional three-lead CRT-D system (e.g., if atrial sensing amplitude measured by a conventional pacing system analyzer was below 1.0 mV) were excluded from the statistical analysis.

Descriptive statistics are expressed as the mean ± standard deviation or median and interquartile range (IQR) for metric variables, and as the count and percentage for categorical variables. Intraindividual changes in ordinal and metric data were analyzed using the non-parametric Wilcoxon signed-rank test. Event-free rates and 95% confidence intervals (CI) for the primary and secondary endpoints were estimated using the Kaplan–Meier method.

All device-based statistics during follow-up were derived from remote monitoring data. Patient-specific virtual follow-up values were simulated by calculating the average of values obtained through daily messages within a 14-day interval around 30 days (1 month), 183 days (6 months), and 365 days (12 months) after implantation if at least two daily messages were transmitted for the given virtual follow-up point. Descriptive statistics were then performed analogously to the data collected via case report forms.

## 3. Results

### 3.1. Patients

Of the 113 patients initially enrolled in the study, 2 were excluded due to failed placement of the LV lead and 1 was excluded after receiving a device not specified in the study protocol. No patient received an RA lead during initial implantation.

At baseline, the patients were 62 ± 12 years old and 77 (70.0%) were male (Table 1). The underlining heart disease was mainly non-ischemic (60.0%), and the mean LVEF was 26 ± 7%. Although the majority of patients (93.6%) received beta-blockers, the mean heart rate at rest was as high as 69 ± 12 bpm. The maximum heart rate at exercise was 116 ± 17 bpm, which was determined through an exercise test (49.5%), 6 min walk test (29.4%), Holter ECG (11.9%), or other ECG methods (9.2%).

### 3.2. Follow-Up

Of the 110 study patients, 104 were followed for 12 months and 6 were terminated from the study earlier: 1 due to CRT-DX system explantation caused by infection, 2 due to deaths unrelated to the study intervention (car accident and pneumonia), 2 who withdrew informed consent, and 1 was lost to follow-up. The mean follow-up period was 363 ± 68 days.

### 3.3. Primary Endpoint: Implantation of an RA Lead

Two patients required late RA lead implantation during the observation period. The first patient, initially meeting the inclusion criteria with a resting heart rate of 53 bpm and maximum rate of 107 bpm, developed chronotropic incompetence. Remote monitoring showed a decline in daily mean heart rate from 59 bpm in the first 2 post-operative months to 55 bpm before RA lead implantation at 7.5 months (day 229) post-CRT-DX implantation.

The second patient experienced immediate post-implantation atrial undersensing, with only five valid atrial amplitude measurements (0.6–0.9 mV) in the first month and no subsequent improvement, leading to RA lead implantation at 6.5 months (day 213). This patient’s initial eligibility for CRT-DX was uncertain due to undocumented atrial sensing amplitudes measured by an external pacing system analyzer during implantation, which should have excluded patients with amplitudes < 1.0 mV.

These two cases resulted in an RA lead implantation-free rate at 365 days of 98.2% (CI: 92.7–99.5%).

### 3.4. Secondary Endpoints

During follow-up, 11 surgical re-interventions related to any component of the CRT-DX system were carried out in 11 patients (10%), which corresponds to an event-free rate of 89.9% at 12 months (CI: 82.4–94.3%). Nine of these surgical re-interventions were related to leads, including two cases of RA lead implantation, three LV lead dislodgements, two RV-DX lead dislodgments, one RV-DX threshold increase, and one pocket infection followed by CRT-DX system explantation (Table 2). The 12-month event-free rate for lead-related re-interventions was 91.7% (CI: 84.6–95.6%), and for pocket infections, it was 99.1% (CI: 93.6–99.9%). The two surgical re-interventions unrelated to leads involved a healing disorder without evidence of infection and device repositioning due to shoulder pain and arm numbness. No lead perforation was reported.

### 3.5. Atrial Sensing and Atrio-Biventricular Pacing

**Figure 1:**
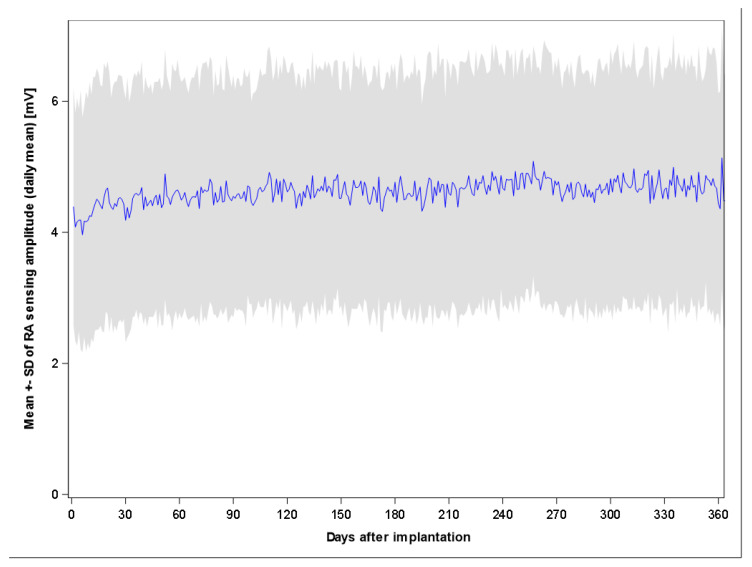
Daily mean RA sensing amplitude (blue graph) continuously documented by remote monitoring in 102 patients with CRT-DX configuration throughout the 12-month follow-up period. CRT-DX = cardiac resynchronization therapy defibrillator with atrial diagnostic dipole. RA = right atrial.

In the 102 patients who remained in the CRT-DX configuration until the study’s end (excluding 2 patients who transitioned to conventional three-lead CRT-D after RA lead implantation from the original cohort of 104 that was followed for 12 months), the mean daily RA sensing amplitudes, measured remotely, were 4.7 ± 1.7 mV at 1 month, 4.9 ± 1.8 mV at 6 months, and 4.7 ± 1.7 mV at 12 months. The corresponding mean daily minimum values were 4.0 ± 1.7 mV, 4.1 ± 1.7 mV, and 4.0 ± 1.8 mV, respectively. As illustrated in Figure 1, the RA sensing amplitude remained stable within the observational time.

Comparatively, the mean intraoperative RA sensing amplitude measured by the implanted CRT-DX at the final lead position was 6.8 ± 4.4 mV (median: 5.8 mV; IQR: 4.0–8.7). When measured using an external pacing system analyzer—without the advanced signal processing and amplification provided by the CRT-DX—the mean amplitude was 2.8 ± 2.4 mV (median: 2.0 mV; IQR: 1.4–3.0). Notably, the RA sensing amplitudes measured by the external pacing system analyzer were <1.0 mV in four enrolled patients, while data were unavailable for five patients.

The median value of the atrial sensing-triggered ventricular pacing (AS-VP) or atrial sensing followed by intrinsic rhythm (AS-VS) was 99.3% (IQR: 97.8–99.9) at 1 month, 99.4% (IQR: 97.6–100) at 6 months, and 99.8% (IQR: 98.2–100) at 12 months. The median percentage of biventricular pacing was 98.8% (IQR: 94.9–99.8), 98.9% (IQR: 95.1–99.9%), and 98.5% (IQR: 94.6–100), respectively.

### 3.6. Clinical CRT Response

**Figure 2:**
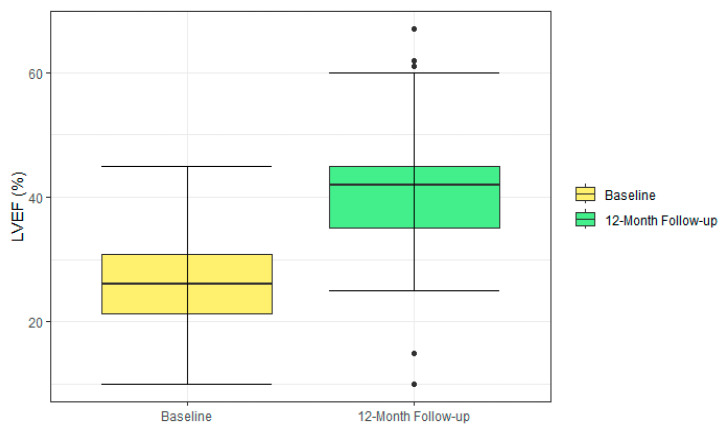
Boxplot of LVEF values measured at baseline and after 12 months of CRT-DX therapy in 95 patients with paired data. The central line within each box represents the median, the box boundaries denote the interquartile range (IQR), whiskers extend to the smallest and largest values within 1.5 times the IQR from the quartiles, and circles represent the individual values outside the whiskers. CRT-DX = cardiac resynchronization therapy defibrillator with atrial diagnostic dipole, LVEF = left ventricular ejection fraction.

Paired echocardiographic data from baseline (without CRT) and the study end in the CRT-DX configuration were available for 95 patients. The mean LVEF increased significantly by 14.7% ± 11.0%, rising from 26.1% ± 6.7% at baseline to 40.8% ± 10.7% at the study’s end (*p* < 0.001, Figure 2).

**Figure 3:**
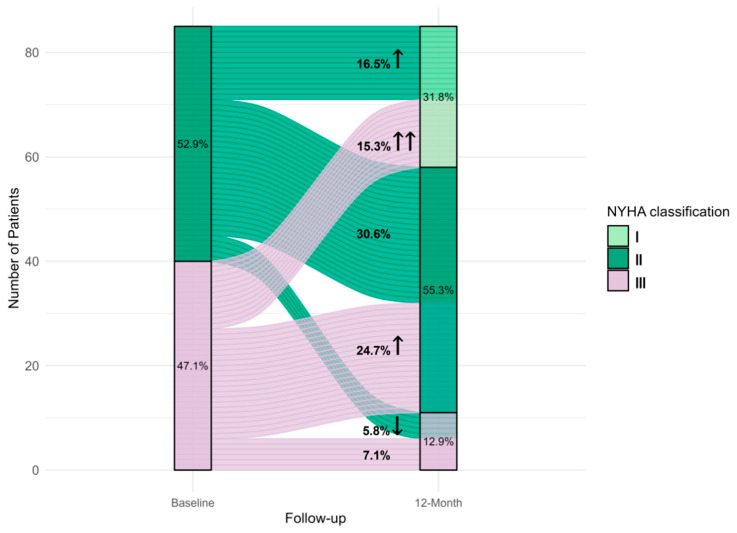
Sankey diagram for the change in NYHA functional class after 12 months of follow-up compared to baseline in 85 patients with paired data. Upward arrows indicate improvement (decrease) in the NYHA class. Downward arrows indicate worsening (increase) in the NYHA class. NYHA = New York Heart Association.

NYHA class assessments at baseline and the study end in the CRT-DX configuration were available for 85 patients. Among these, 48 patients (56.5%) demonstrated improvement by one (35 patients, 41.2%) or two (13 patients, 15.3%) classes. No changes were observed in 32 patients (37.6%). Five patients (5.8%) experienced a worsening by one class (global *p*-value < 0.001, Figure 3).

## 4. Discussion

In this study of 110 CRT-D patients in sinus rhythm who received a CRT-DX system without an atrial lead, only two cases required the addition of an atrial lead during the first year of follow-up. Before implantation, chronotropic competence was assessed using Holter ECG monitoring, a 6 min walk test, or an ergometer test, with patients excluded if their maximum achieved heart rate did not exceed 100 bpm. These findings suggest that the CRT-DX concept may be clinically applicable using a simple decision algorithm.

The BIO|REDUCE study adds data on the use of CRT-DX systems in a standard care setting, while previous studies focused on proof-of-principle and sub-analyses of a study primarily targeting left ventricular lead performance [10,11].

Reducing the complexity of implantable therapeutic devices can simplify their use, lower complication rates, and mitigate costs. In the context of CRT, omitting the atrial lead in patients with sinus rhythm and preserved chronotropic competence is feasible if an atrial dipole on the RV lead ensures AV synchrony. Several recent studies suggested that CRT-DX systems are safe and reliable [8,10,11]. However, concerns remain regarding optimal patient selection, and additional data on the stability of atrial signals and CRT responses with CRT-DX systems would further support their broader clinical adoption.

The BIO|REDUCE study applied the simple clinical selection criteria of a sinus rate > 40 bpm at rest and ≥100 bpm during exercise under individually optimized pharmacological heart failure therapy, combined with the absence of persistent atrial fibrillation or bradycardia–tachycardia syndrome. The need for atrial pacing and subsequent transition to a conventional CRT-D—requiring an additional implantation procedure—was 1.8% during the 12 months of follow-up. In two earlier studies with a total of 157 patients, no patient needed an RA lead implantation was found for the CRT-DX system over more than one year of follow-up [10,11]. This suggests that the RA lead implantation rate is lower than the typical atrial lead re-intervention rates using conventional CRT-D systems [11,15,16,17,20,21,22], although a randomized comparison would be required for a conclusive answer. Notably, one of the two patients requiring an additional atrial lead implantation had received the CRT-DX system despite exhibiting low atrial signal amplitudes during early follow-up—likely detectable at the time of implantation but neglected by the implanter in deviation from protocol recommendations. This underscores the critical importance of strict adherence to CRT-DX selection criteria, which could minimize the need for RA lead upgrades when rigorously applied.

Patients with frequent premature ventricular complexes and those with second- or third-degree AV block were excluded from the CRT-DX study to optimize the data quality of AV-synchronous pacing. These study-related exclusion criteria should not be interpreted as clinically significant restrictions on the use of the CRT-DX system in routine practice.

As secondary endpoints, device- and lead-related re-interventions demonstrated an overall re-intervention rate of 10.0% (only 8.2% were lead-related), including the two patients (1.8%) who reached the primary endpoint of atrial lead implantation. Overall, he lead-related complication rate was comparable to that in previous reports for conventional CRT-D systems [21,22,23], which is consistent with prior CRT-DX studies that also reported device-related re-intervention profiles similar to those with conventional CRT-D systems [10,11].

Overall, a high rate of atrio-biventricular synchronous pacing (>98%) was observed in the BIO|REDUCE study, as would be expected for conventional CRT-D systems equipped with an RA lead, and it meets the expectation to be as high as possible [2,24]. This high biventricular pacing percentage during follow-up is attributable to the stable atrial signal amplitudes, which averaged 4.7 mV throughout the study. Variations in signal amplitudes were noted; however, the minimum values derived from the daily data transmissions were well above the margin required to ensure reliable atrio-biventricular pacing. Furthermore, these findings suggest that atrial signal quality is also adequate for the detection of atrial tachyarrhythmias in this patient population [8]. The results of the present study highlight the efficacy of the CRT-DX system’s advanced signal processing algorithm and align with those of previous investigations showing stable and adequate atrial sensing across different body positions over time in single-chamber systems [25].

The relatively large post-processed atrial signal amplitudes should not lead to the assumption that any signal can be appropriately processed. There is a lower threshold below which signal processing becomes challenging for the algorithm. In this study, a cutoff of 1.0 mV for atrial signal amplitude, measured using a standard pacing system analyzer, was recommended for lead position acceptance. This threshold was not met in four patients, one of whom experienced atrial undersensing of sinus rhythm during follow-up. Therefore, the value of 1.0 mV seems to be a threshold that should not be undercut, and it is advisable to achieve larger signal amplitudes.

Adequate atrial signals for atrio-biventricular synchronous pacing are a pre-requisite for a CRT response. In this study, CRT responses were measured as changes in LVEF and NYHA class between study inclusion and the 12-month follow-up. A statistically significant increase in LVEF by a mean of 14.7% was observed in the patients with paired data available at both time points, documenting a robust response to CRT with a well-functioning CRT-D system. Large CRT trials typically report an absolute increase in LVEF of approximately 4–11% [4,26,27]. Whether the greater improvement observed in our study reflects the patient selection criteria, such as chronotropic competence during sinus rhythm as a surrogate for less advanced heart disease, or is due to random effects related to the limited study size remains uncertain.

Corresponding to the significant increase in LVEF observed in our study cohort, the NYHA class showed both clinical and statistically significant improvements. More than half of the patients achieved an improvement of at least one NYHA class, while CRT non-response or disease progression were infrequent.

Omitting the atrial lead is not the only emerging strategy for hardware simplification in CRT. Recent advancements have introduced dual-chamber CRT devices with ventricular leads positioned at the His bundle or left bundle branch area to implement conduction system pacing (CSP). Non-randomized studies have shown very promising effects of CSP on heart failure management in patients with traditional CRT pacemaker indications and in preventing heart failure when ventricular antibradycardia pacing is required [22,28,29,30]. These encouraging findings have been further supported by more recent randomized trials [31,32,33]. Currently, CSP primarily targets pacemaker recipients, while the DX system addresses the population for cardioverter–defibrillator implantation. In the future, merging the two trunks of simplification—CRT via CSP and atrial lead omission through a DX system—could pave the way for a single-lead CRT-D system [34].

### Limitations of the Study

The BIO|REDUCE study was designed as an observational investigation with a limited number of patients recruited during the COVID-19 pandemic. The study provides data for up to 12 months after CRT-DX implantation but lacks long-term follow-up results. Interpretation of the findings requires comparison with historical cohorts due to the absence of a control group. Despite these limitations, the study offers valuable insights into patient selection for CRT-DX systems and demonstrated CRT response rates that are comparable to those reported in previous studies with traditional CRT-D systems. The study provides a basis for a future randomized trial that directly compares CRT-DX with traditional three-lead CRT-D systems.

## 5. Conclusions

When clinically practical and simple inclusion criteria are used, the need for subsequent RA lead implantation in patients with two-lead CRT-DX systems (1.8%) may be lower than the RA lead complication rate observed in conventional three-lead CRT systems. Atrial sensing proved effective in maintaining reliable AV synchrony and achieving a high proportion of biventricular pacing. The two-lead CRT-DX concept may be a feasible alternative for patients with preserved chronotropic competence.

## Figures and Tables

**Table 1:** Patient characteristics at baseline.

Characteristic	Value (*n* = 110)
Age (years)	62 ± 12
Male/female sex	77/33 (70.0/30.0)
Primary prevention of sudden cardiac death	97 (88.2)
Non-ischemic cardiomyopathy	66 (60.0)
LVEF (%)	26 ± 7
NYHA class I II III IV	0 (0) 60 (54.5) 50 (45.5) 0 (0)
Left bundle branch block	103 (93.6)
Atrioventricular block None I° II°–III°	96 (87.3) 14 (12.7) 0 (0)
History of paroxysmal atrial fibrillation	13 (11.8)
Heart rate at rest (bpm), *n* = 109	69 ± 12
Maximum heart rate at exercise (bpm), *n* = 109	116 ± 17
QRS duration (ms)	161 ± 23
Concomitant diseases Diabetes mellitus Renal failure Hypertension	38 (34.5) 16 (14.5) 73 (66.4)
Medication	
Beta-blocker	103 (93.6)
ACE inhibitor/ARB/ARNI	101 (91.8)
Aldosterone antagonist	90 (81.8)
Diuretic	69 (62.7)
Glycoside	3 (2.7)

Data are shown as mean ± standard deviation or *n* (%). ACE = angiotensin converting enzyme, ARB = angiotensin II receptor blocker, ARNI = angiotensin receptor/neprilysin inhibitor, bpm = beats/minute, LVEF = left ventricular ejection fraction, NYHA = New York Heart Association.

**Table 2:** Complication rates and mortality after 12 months of follow-up in 110 patients initially implanted with CRT-DX.

Complication			Patients with Events, *n* (%)	Events, *n*	Event-Free Rate for Primary and Secondary Endpoints, % (95% CI)
Perforation			0 (0)	0	
Pneumothorax with drainage			3 (2.7)	3	
Post-operative system revision with invasive re-intervention			11 (10.0)	11	89.9 (82.4–94.3) ^b^
	Lead complication with surgical re-intervention		9 (8.2)	9	91.7 (84.6–95.6) ^b^
		RA lead implantation after successful initial implantation of the CRT-DX system	2 (1.8)	2	98.2 (92.7–99.5) ^a^
		RV-DX lead dislodgment	2 (1.8)	2	
		RV-DX lead threshold increase	1 (0.9)	1	
		LV lead dislodgment	3 (2.7)	3	
		Pocket infection with CRT-DX explantation	1 (0.9)	1	99.1 (93.6–99.9) ^b^
	Wound healing disorder		1 (0.9)	1	
	CRT-DX repositioning due to pain/numbness		1 (0.9)	1	
All-cause mortality			2 (1.8)	2	
	Cardiovascular mortality		0 (0)	0	

CI = confidence interval for event-free rates estimated using the Kaplan–Meier method, CRT-DX = cardiac resynchronization therapy defibrillator with atrial diagnostic dipole, LV = left ventricular, RA = right atrial, RV-DX = right ventricular lead with atrial sensing dipole and defibrillation coil. ^a^ referring to primary endpoint defined as an additional RA lead implantation, ^b^ referring to secondary endpoints defined as (i) any post-implant surgical re-intervention involving any component of the CRT-DX system, (ii) any lead complication, and (iii) device or pocket infection.

## Data Availability

The data underlying this article will be shared upon reasonable request to the corresponding author due to privacy reason.
